# Emergency Medical Services Professionals’ Attitudes About Community Paramedic Programs

**DOI:** 10.5811/westjem.2017.3.32591

**Published:** 2017-05-01

**Authors:** Robert J. Steeps, Denise A. Wilfong, Michael W. Hubble, Daniel L. Bercher

**Affiliations:** * Northwest Arkansas Community College, Division of Health Professions, Emergency Medical Sciences Program, Bentonville, Arkansas; † Western Carolina University, School of Health Sciences, Emergency Medical Care Program, Cullowhee, North Carolina; ‡ University of Arkansas for Medical Sciences, College of Health Related Professions, Department of Emergency Medical Services, Little Rock, Arkansas

## Abstract

**Introduction:**

The number of community paramedic (CP) programs has expanded to mitigate the impact of increased patient usage on emergency services. However, it has not been determined to what extent emergency medical services (EMS) professionals would be willing to participate in this model of care. With this project, we sought to evaluate the perceptions of EMS professionals toward the concept of a CP program.

**Methods:**

We used a cross-sectional study method to evaluate the perceptions of participating EMS professionals with regard to their understanding of and willingness to participate in a CP program. Approximately 350 licensed EMS professionals currently working for an EMS service that provides coverage to four states (Missouri, Arkansas, Kansas, and Oklahoma) were invited to participate in an electronic survey regarding their perceptions toward a CP program. We analyzed interval data using the Mann-Whitney *U* test, Kruskal-Wallis one-way analysis of variance, and Pearson correlation as appropriate. Multivariate logistic regression was performed to examine the impact of participant characteristics on their willingness to perform CP duties. Statistical significance was established at p ≤ 0.05.

**Results:**

Of the 350 EMS professionals receiving an invitation, 283 (81%) participated. Of those participants, 165 (70%) indicated that they understood what a CP program entails. One hundred thirty-five (58%) stated they were likely to attend additional education in order to become a CP, 152 (66%) were willing to perform CP duties, and 175 (75%) felt that their respective communities would be in favor of a local CP program. Using logistic regression with regard to willingness to perform CP duties, we found that females were more willing than males (OR = 4.65; p = 0.03) and that those participants without any perceived time on shift to commit to CP duties were less willing than those who believed their work shifts could accommodate additional duties (OR = 0.20; p < 0.001).

**Conclusion:**

The majority of EMS professionals in this study believe they understand CP programs and perceive that their communities want them to provide CP-level care. While fewer in number, most are willing to attend additional CP education and/or are willing to perform CP duties.

## INTRODUCTION

The number of patients presenting to emergency departments (ED) in the United States has been steadily increasing over the past three decades.^1^ The Centers for Disease Control and Prevention estimated the total number of ED visits in 2009 to be 136.1 million,^1^ a 16.5% increase from the estimated 116.8 million ED visits in 2007.^1^ With the increase in annual ED visits, the time a patient has to wait to see a provider has also increased 25% from 46.5 minutes to 58.1 minutes between 2003 and 2009.^2^ America’s emergency medical services (EMS) systems and EDs alike have experienced the increased demands to treat patients seeking care.^3^ One of several approaches to address this strain on emergency services is a new model of care provided by community paramedics (CP).^3^

The traditional model of care used by EMS systems in the U.S. has been to treat and transport patients to the ED for further assessment and care provided by physician and nursing staff. While each community’s respective CP program can be tailored to its individual needs, the CP model, in general, uses specially educated paramedics to treat minor injuries and manage chronic illnesses in the patient’s home and/or arrange care provided in the community, thereby limiting unnecessary transports to the ED.^3^,^4^ By reducing the number of patients transported to the ED, this model of care has the potential to lessen the burden on both transport ambulances and EDs while providing an improved experience for patients by avoiding long ED wait times.^3^

Potential benefits of patients being assessed and treated in the prehospital environment include a reduction on the patient load in already strained EDs and a reduction in cost along the healthcare continuum. As the Affordable Care Act implementation continues to unfold, reducing the number of patients readmitted to the hospital within 30 days of discharge with diagnoses such as congestive heart failure, acute myocardial infarction, and pneumonia will also be financially advantageous for hospitals faced with potentially reduced levels of reimbursement for services.^5^

Although the problem of ED crowding from increased patient visits has been identified, and some local CP programs have demonstrated a reduction in the number of patients transported to these EDs,^6^–^9^ the challenges of implementing and staffing CP programs need to be addressed. These challenges include cost, availability of appropriate education programs for potential providers, support from the local community, a reliable reimbursement stream, and acceptance from EMS professionals. Of these, attaining buy-in from EMS professionals is paramount, yet this precursor to successful implementation has not been evaluated. The goal of this study was to investigate EMS professionals’ attitudes about and willingness to participate in CP programs.

**Population Health Research Capsule**

What do we already know about this issue?

Several studies have been published regarding community paramedic (CP) programs; we have not found any that address EMS professionals’ willingness to participate.

What was the research question?

What are EMS professionals’ attitudes about CP programs and are they willing to participate in them?

What was the major finding of the study?

Most EMS professionals stated they were willing to participate in a CP program.

How does this improve population health?

Meeting patients’ healthcare needs in their respective communities may reduce unnecessary ED visits and lessen the strain on crowded EDs.

## METHODS

### Study Design

The authors used a cross-sectional survey (Supplement) method in this evaluation. Despite a thorough review of the literature, the authors were unable to identify a specific study instrument that addresses EMS professionals’ perceptions of a CP program. Therefore, the survey instrument design was adapted from one used by Bercher in his evaluation of the attitudes of paramedics toward the performance of home hazard inspections in addition to their routine daily work tasks.^10^

### Study Setting

EMS professionals (EMTs [emergency medical technicians] and paramedics) practicing in a hospital-based Advanced Life Support (ALS) EMS service headquartered in southwest Missouri were used as a sample of convenience. This EMS service delivers ALS ground coverage for 15 counties in four states (Missouri, Arkansas, Kansas, and Oklahoma) and consists of 9,871 square miles of service area. The coverage area is a mixture of rural, urban, and suburban areas with a total population of 686,462. The annual call volume is over 60,000 ambulance requests covered by 350 EMS professionals working out of 29 stations with 48 ambulances.

### Study Sample and Demographic Variables

To acquire a representative sample from the entire coverage area of the service, a request for voluntary participation by EMS professionals and a link to the electronic survey were sent to their respective hospital-issued electronic mail address by each regional manager. We collected demographic information (age, gender, race, education level, current level of EMS licensure, years of EMS service, average number of calls per shift, typical length of shift, type of EMS service, current rank, and type of community served) via the survey instrument. The survey was delivered in an electronic format using Qualtrics online software.^11^

### Survey Design and Validation

We asked participants to respond to a series of statements using a seven-point Likert-type scale. The survey was validated using an expert panel of four individuals with experience in emergency medical care. Three of the evaluators are EMS professionals with experience and licensure as paramedic-level instructors. Two have doctoral degrees, one of whom developed a CP education program at his university. The final evaluator is a practicing emergency physician with 19 years of experience and currently serves as the medical director for an ED, two EMS services, and an EMS education program. Following review and revision by the expert panel members, a pilot of the revised instrument was performed.

The pilot testing sample consisted of a group of 16 currently licensed and practicing EMS professionals. We obtained a written letter of agreement to recruit participants from the service’s chief officer prior to initiation of the survey. These individuals were requested to provide additional feedback on ease of use, readability, and offer suggestions for further improvements. The pilot group was excluded from participating in the study.

### Study Participant Protection

The Western Carolina University Institutional Review Board (IRB) approved this study. It also received approval from the IRB representing the hospital that oversees the EMS service which participated in this study. All study subjects granted their consent prior to participation in the study, and their responses were anonymous.

### Data Analyses

All data were uploaded from Qualtrics into and analyzed with the Statistical Package for the Social Sciences (SPSS).^12^ We used descriptive statistics to summarize participants’ responses as means, medians, and percentages with regard to subjects’ demographics. Percentages were calculated based on the total number of respondents to each respective question. Kolmogorov-Smirnov tests of normality indicated that the data were not normally distributed. Consequently, we used nonparametric testing. The alpha level for each of the statistical evaluations was set at p ≤ 0.05.

We used a Mann-Whitney *U* test to analyze the effects of gender as well as the current usage of a CP model concerning participants’ willingness to perform CP duties. A Kruskal-Wallis one-way analysis of variance was used to analyze the effects of participant characteristics (including EMS provider level, education level, type of EMS shift, community served, current rank, and perceived hours per shift that could be dedicated to CP duties) on their outcomes. We used the Pearson product-moment coefficient of correlation to determine if there was a relationship between factors (years of EMS experience, number of patient calls per shift, and age) and participant outcomes. Multivariate logistic regression was also performed to examine the impact of participant characteristics (such as gender, age, race, EMS provider level, level of education, type of shift, community served, current rank, and perceived hours per shift dedicated to CP duties) on the likelihood of participants’ willingness to perform CP duties.

## RESULTS

### Survey Response

The survey was opened on January 19, 2015, and closed on February 23, 2015. Members of the EMS service used for this research were notified of survey availability via an email to their work email account sent from their manager. Of the 350 EMS professionals receiving an invitation, 283 (81%) participated. Consent was given by 277 (98%) of the respondents and comprised the final data set for analysis. There were no sequential questions that forced participants to respond to a question before answering additional questions. Consequently, not all questions were answered by all respondents. See Table 1 for sample demographic characteristics.

### EMS Work Experience

While the survey was conducted using a hospital-based EMS service, many of its part-time coworkers have primary EMS employment with different types of services. As expected, hospital-based EMS was the type of service listed as the primary work experience for a majority of the participants (198, 83%). Survey responses were representative of differing types of response settings and hours worked per shift (Table 2).

### Perceived CP Needs

When questioned about the perceived number of hours per shift worked in which the participant could commit to a CP program, 73 (31%) indicated that they could not commit any time. A majority of the respondents (162, 69%) stated that they could spend one hour or more, with 51 (22%) indicating that they could commit more than four hours per shift to CP duties. Providers perceived that 47% (SD 1.7) of the patients they currently encounter in the field could potentially benefit from a CP program.

### CP Attitudinal Responses

When questioned as to their confidence in their understanding of what a CP program entails, 165 (70%) revealed feeling knowledgeable as to the requirements of a CP program. Eighteen (8%) selected a neutral response. Fifty-three (22%) indicated that they did not have a good understanding about CP programs. Regarding EMT and paramedic respondents’ perceived understanding of CP programs, positive responses were given by 49 (65%) and 111 (75%) respectively. A total of 135 (58%) survey participants indicated that they were likely, somewhat likely, or very likely to attend additional education to become a CP with 45 (19%) undecided. Fifty-six (23%) indicated that they were unlikely, somewhat unlikely, or very unlikely to participate in additional CP education.

In response to whether or not they would perform CP duties with as much or more enthusiasm as they currently have for traditional, prehospital patient care, 152 (66%) survey participants indicated that they would. The number of undecided was 38 (16%). Forty-three (18%) gave a negative response. Regarding responses per licensure level, 44 (58%) EMTs and 102 (69%) paramedics indicated a willingness to participate in a CP program.

When questioned if a CP program should be a significant responsibility for EMS in their community, 174 (74%) respondents gave a positive indication. Thirty-eight (16%) were neutral in their responses. The 24 (10%) other respondents perceived that a CP program should not be a significant responsibility for their community’s EMS agency.

Three-fourths of those participating (175, 75%) felt that their respective community would be in favor of their service performing CP duties. Forty-eight (21%) were unsure of their community’s reaction to a local CP program. A minority of the respondents (11, 4%) felt that their community would not be in favor of CP duties being performed by their service. See Table 3 for further explanation of participant responses.

### Data Analyses

Regarding the respondents’ type of community served and their willingness to complete CP duties, we found no statistical significance (p = 0.74). Participants who reported working for an EMS service that currently uses a CP model of patient care delivery did not have a statistically significant difference (p = 0.89). Additionally, reported rank (field provider of care vs. non-field provider of care [supervisor]) and the participants’ willingness to perform CP duties did not produce a statistical significance (p = 0.34). See Table 4 for further explanation of the data analyses.

Regarding participants’ willingness to perform CP duties, the results of the regression model correctly classified 79.2% of all the cases. Females were four times more likely than males to indicate a willingness to perform CP duties (OR = 4.651, p = 0.03; 95% CI 1.186, 18.236). The respondents perceiving that they had no spare time while on duty to commit to CP duties were less willing to accept the additional duties of a CP program than those who perceived they had any time on duty for these activities (OR = 0.198, p < 0.001; 95% CI .087, 0.449). See Table 5 for further logistic regression results.

## DISCUSSION

While some reports on the effectiveness of CP programs regarding the reduction of ED bed hours, unnecessary ambulance transports, and emergency services’ cost savings appear in the literature,^1^,^3^,^6^ the authors were unable to identify any studies on the attitudes of EMS professionals toward their understanding of these programs or their willingness to participate in them. Our survey findings indicate that EMS professionals believe that they have an understanding of CP programs and most are willing to volunteer to attend additional education in order to participate in them. EMS professionals also feel that CP programs will help those in their community who have the greatest need and that CP programs should be a significant responsibility for EMS in their respective communities. Responses also indicate that most, but not all, EMS professionals are willing to perform CP duties with as much or more enthusiasm as they currently have for traditional, prehospital patient care. However, the time commitment for such duties was a concern, suggesting that successful implementation may be more likely when staff members are committed directly to CP duties instead of dual responsibilities.

Female participants were more than four times as likely as their male cohorts to express a willingness to participate in a CP program. Female willingness may be impacted, in part, by participants’ empathy levels. Compared to males, Williams et al. found that female paramedic students had higher empathy ratings toward all medical conditions queried.^13^ Similar results were reported by other authors evaluating healthcare students’ empathy levels.^14^–^17^

We found no statistically significant differences in willingness to participate in a CP program with regard to EMS provider level, age, level of education, type of shift, community served, or current rank. EMTs and paramedics both expressed an interest in providing CP-level care to their communities. While a study by Simpson et al. found that younger EMS providers with a tertiary education had a higher level of support for evidence-based practice,^18^ young EMS professionals were as likely as their older counterparts to express a willingness to participate in a CP program. In addition, educational preparation, shift hours worked, and rural vs. urban practice settings were not found to impact EMS professionals’ willingness to participate in a CP program.

### Attitudinal Implementation Barriers: Parallels with other Public Safety Professions

Prior to implementing a CP program, EMS leaders should investigate potential barriers to successful implementation. While there is a paucity of research exploring the attitudes of EMS professionals regarding CP programs, the importance of employee acceptance to successful implementation of new programs has been reported among other public safety professions. For example, several reports examining the attitudes of law enforcement officers (LEOs) and firefighters toward the nontraditional role of providing care to patients in the prehospital environment exist in the literature.^19^ Such reports may provide insights and parallels to gauging EMS professionals’ attitudes toward implementing CP programs.

Over the past four decades, policing strategies have changed from an enforcement role to one of problem-solving.^20^ These new roles have included the use of automated external defibrillators (AEDs) and cardiopulmonary resuscitation (CPR) for victims of out-of-hospital cardiac arrest, as well as officer-administered naloxone to treat overdose victims.^20^ With CP programs changing the EMS profession from a primarily reactive role to one of prevention, EMS leadership might encounter similar implementation barriers faced by their law enforcement counterparts.

Green et al. found that LEOs expressed concerns with the added responsibilities inherent in preventing overdoses in a naloxone administration program.^20^ With the new focus on assessing and managing patients with chronic medical conditions in their respective communities, EMS professionals may also have concerns about the added responsibilities of CP programs. One-fourth (27%) of those participating in our survey perceived that they became an EMS professional to respond to emergency calls and not to participate in a CP program. These respondents might see the new CP responsibilities as a barrier to participation.

When attempting to implement an AED program, Husain et al. found that law enforcement leaders were challenged by LEOs who did not believe providing medical care was a part of their role in the community.^21^,^22^ The lack of officer comfort with providing medical care in the prehospital environment has been linked to a failure to implement this role change.^21^–^23^ Leaders of EMS programs attempting to develop and/or staff CP programs might be challenged to recruit willing participants if EMS professionals do not believe that CP is part of their role. Forty percent of participants responding to our survey did not perceive that their coworkers would be in favor of performing CP duties. Lack of coworker willingness to participate in this role change is a potential implementation barrier.

Another obstacle faced when attempting to implement law enforcement and fire service AED and naloxone programs was hesitancy of the LEOs and firefighters to use the new equipment out of a concern for perceived new liability.^23^,^24^ Prina et al. found that LEOs and firefighters felt that the public’s perceptions about AED success were unrealistically high.^25^ This also created a fear of liability if their resuscitation attempts were unsuccessful.^25^ With the CP focus of providing and/or arranging care in the patient’s community instead of transporting patients to the ED, liability concerns from EMS professionals may also create a barrier to successful CP program implementations. Nearly three-fourths (74%) of our respondents felt that a CP program should be a significant responsibility for EMS in their respective community. This may indicate that liability concerns are not dissuading our survey participants from expressing a willingness to participate in CP programs.

Many obstacles to successful implementation of novel programs in emergency professions that can correlate to EMS providers’ implementation of CP programs have been identified. Fortunately, several items have been found to assist with the start of new programs. When LEOs and firefighters felt that their new roles could benefit the communities they served, they were more likely to have positive attitudes toward these new roles.^21^,^23^,^25^–^27^ A majority (84%) of our respondents believe that a CP program would help those in their community with the greatest needs. Like their LEO counterparts, the EMS professionals who understand the potential benefits a CP program could offer to the underserved and vulnerable members of their community might be more willing to participate.

All police chiefs and a majority of their respective officers surveyed by Papson et al. believed that the use of AEDs by police was not only appropriate, but also a valuable service to their communities.^23^ A strong show of support from EMS leaders toward CP programs may also translate to increased positive attitudes among their service’s frontline providers. Almost three-fourths (74%) of our respondents felt that their leaders would support a CP program. These positive feelings regarding CP-level care may bode well for successful CP implementation.

In addition to the benefits provided to the community via the new roles of LEOs and firefighters, a majority of public safety professionals surveyed agreed that providing EMS-related activities improved the public’s perception of the participating departments and their members.^23^–^25^,^30^ As EMS professionals transition into the expanded CP roles, this additional service provided to the community may also improve the public’s perception of these providers and their agencies. Of those responding to our survey, three-fourths perceive that the community they serve would be in favor of having a CP program delivered by their EMS agency.

A positive attitude among LEOs and firefighters was found when they personally experienced the impact of EMS-related activities while performing their traditional public service duties.^19^,^20^,^25^ An even higher trend of favorable attitudes was found among those officers and firefighters who restored a pulse for a victim of out-of-hospital cardiac arrest.^25^ Ray et al. found a reluctance, even toward naloxone training, with LEOs who had less experience or lacked recent experiences with overdose cases.^28^ EMS professionals may have a more positive attitude toward CP programs when they can see the tangible benefits to the community they serve. Respondents to our survey felt that nearly half (47%) of the patients they currently encounter in the field could potentially benefit from a CP program.

A significant barrier to implementing AED programs for public safety professionals has been negative attitudes related to a lack of education regarding the programs and their benefits.^29^ To overcome this barrier, educating LEOs and firefighters about these public health programs has shown to increase their willingness to participate.^19^,^23^–^25^,^29^ EMS professionals educated about CP programs and their subsequent benefits to the community may be more willing to participate in them. A majority of our survey respondents (70%) perceived that they currently have a good understanding of CP programs and stated they would volunteer (58%) to attend additional education to become a CP.

EMS leaders interested in pioneering a CP program in their area could use this information when making strategic plans for growth and expansion of their services in the community. This survey’s results indicate that EMS professionals perceive they understand CP programs, support providing CP services to their community, and are willing to participate in this model of care delivery.

## LIMITATIONS

A significant limitation to this study was a lack of diversity among the subjects regarding the type of their primary EMS service. The service used for this study was hospital-based. While the service does employ part-time EMS professionals who have full-time employment at different types of EMS services, a majority of the respondents were hospital-based. The sample size was also relatively small and geographically non-diverse. A majority of the participants also had no current CP experience.

The lack of racial and gender diversity among the participants are other limiting factors. Survey respondents identified themselves as White and as male, 94% and 75% respectively. While a 2013 study by Bentley et al. found that 85% of nationally certified EMS professionals identified themselves as nonminority and 74% as male,^30^ generalization of the findings toward services with differing percentages of ethnicities and genders may be limited.

As with all cross-sectional studies, participants may be biased toward or against participation based on their feelings regarding the topic. In addition, several participants did not respond to all questions. The lack of complete responses combined with self-reporting could have led to biased results.

While our results indicated that EMS professionals are generally receptive to participating in a CP model of care delivery, future studies are needed to confirm the findings in different regions, different types of EMS agencies, and among a more diverse group of EMS professionals.

## CONCLUSION

The authors sought to quantify the attitudes of EMS professionals toward a CP program and found that the majority of those surveyed believe they understand what a CP program entails, most are willing to attend additional education to offer CP services and are willing to serve in this new role. EMS administrators might reach buy-in from their employees if separate CP shifts or CP positions are offered instead of adding these new responsibilities to their employees’ current job duties. Results of this survey are limited by the predominate type of EMS service represented, as well as the geographical location and limited racial and gender diversity among the participants. Further studies are needed to assess the opinions of EMS professionals in differing types of EMS agencies, different geographical locations, and differing proportions of ethnicities and genders. These results will be important for EMS administrators and medical directors planning to develop and implement CP programs.

## Supplementary Information



## Figures and Tables

**Table 1:** Participants’ demographic characteristics in a study of emergency medical services professionals’ attitudes toward community paramedic programs.

Demographics	
Age	
Mean	37 years
Standard deviation	±10.1 years
Gender	
Male	202 (75%)
Female	68 (25%)
Ethnicity	
White	253 (94%)
Hispanic	3 (1%)
American Indian/Alaska Native	6 (2%)
Multi-cultural	4 (1%)
Other	2 (1%)
Education	
Some college	118 (44%)
Associate	59 (22%)
Bachelor	59 (22%)
Master	9 (3%)
Doctorate	3 (1%)
Current level EMS certification/licensure	
EMT	84 (33%)
AEMT	10 (4%)
Paramedic	160 (62%)
Total years EMS experience	
Mean	13 years
Range	0–41 years
Standard deviation	± 0.6
Duration of current level EMS certification/licensure	
Mean	13 years
Range	0–41 years
Standard deviation	± 0.6

*EMS*, emergency medical services; *EMT*, emergency medical technician; *AEMT*, advanced emergency medical technician.

**Table 2:** Participants’ work experiences.

EMS work experience	
Primary EMS work experience	
Hospital-based	198 (83%)
Fire-based	14 (6%)
Private service	13 (5%)
Third/government	8 (3%)
Public utility/nonprofit	6 (3%)
Other	1 (0%)
Typical hours worked per shift	
24 hours	122 (47%)
8–12 hour days	55 (21%)
8–12 hour evenings	7 (3%)
8–12 hour nights	33 (13%)
> 24 hours	33 (13%)
Other	9 (3%)
Type of community served (population size)	
<2,500	30 (13%)
2,500–74,999	119 (50%)
75,000–149,999	15 (6%)
150,000–499,999	64 (27%)
Other	12 (5%)
Typical number of calls/runs per shift worked	
Mean	6
Median	5
Mode	4
Standard deviation	± 0.2
Participants working for a service that currently utilizes community paramedic model	
Yes	13 (6%)
No	222 (94%)
Current rank/position	
Field provider of patient care	166 (69%)
Other (supervisor, manager, dispatcher)	73 (31%)

*EMS*, emergency medical services.

**Table 3:** Participant survey response summary regarding EMS professionals’ attitudes toward community paramedic programs.

Survey question/statement	Somewhat agree, agree, or strongly agree	Neutral	Somewhat disagree, disagree, or strongly disagree
I currently have a good understanding of a CP program.	165 (70%)	18 (8%)	53 (22%)
I would volunteer to attend additional education to become a CP.	135 (58%)	45 (19%)	56 (23%)
A CP program will help those in most need (i.e. the very young, the very old, and the disabled).	197 (84%)	22 (9%)	14 (7%)
A CP program should be a significant responsibility for EMS in my community.	174 (74%)	38 (16%)	24 (10%)
I would perform the duties of a CP with as much or more enthusiasm as I currently have for traditional, prehospital patient care.	152 (66%)	38 (16%)	43 (18%)
My coworkers would be in favor of performing CP duties.	140 (60%)	55 (24%)	39 (16%)
The community I serve would be in favor of our service performing CP duties.	175 (75%)	48 (21%)	11 (4%)
The leaders in my EMS service, in general, would support our organization’s involvement in a CP program.	172 (74%)	35 (15%)	27 (11%)
I became an EMS professional in order to save lives during emergencies - not to participate in a CP program.	64 (27%)	60 (26%)	110 (47%)
My EMS service is not busy enough to benefit from a CP program.	19 (8%)	38 (16%)	176 (76%)
My EMS service is too understaffed to develop a CP program.	98 (42%)	53 (24%)	80 (34%)
Performing CP duties would take up valuable down-time that I depend upon (i.e. for rest and other personal activities).	75 (32%)	64 (28%)	92 (40%)
I work hours that would not be compatible with CP duties for many people.	67 (28%)	67 (28%)	97 (44%)
My EMS service would be willing to develop a specific position or positions dedicated to performing CP duties.	131 (57%)	73 (32%)	27 (11%)

*CP*, community paramedic; *EMS*, emergency medical services.

**Table 4:** Project data analyses.

Dependent variable	Independent variable	p-value
Willingness to perform CP duties	Gender	0.03*
Willingness to perform CP duties	Perceived hours worked per shift dedicated to CP duties	< 0.001†
Current understanding of what a CP program entails	Perceived hours worked per shift dedicated to CP duties	0.01†
Willingness to volunteer to attend additional education to become a CP	Perceived hours worked per shift dedicated to CP duties	< 0.001†
An effective CP program will help those most in need	Perceived hours worked per shift dedicated to CP duties	< 0.001†
A CP program should be a significant responsibility for EMS in my community	Perceived hours worked per shift dedicated to CP duties	< 0.001†
My coworkers would be in favor of performing CP duties	Perceived hours worked per shift dedicated to CP duties	0.01†
The community I serve would be in favor of my service performing CP duties	Perceived hours worked per shift dedicated to CP duties	0.02†
I became an EMS professional in order to save lives during emergencies and not to participate in a CP program	Perceived hours worked per shift dedicated to CP duties	0.01†
My EMS service is too understaffed to develop a CP program	Perceived hours worked per shift dedicated to CP duties	0.02†
I work hours that would not be compatible with CP duties for many people	Perceived hours worked per shift dedicated to CP duties	0.00†
My EMS service would be willing to develop a specific position or positions dedicated to performing CP duties	Perceived hours worked per shift dedicated to CP duties	0.04†
The leaders in my EMS service would support our organization’s involvement in a CP program	Rank	0.05†
I became an EMS professional in order to save lives during emergencies and not to participate in a CP program	Rank	0.02†
I work hours that would not be compatible with CP duties for many people	Typical shift worked	< 0.001†
My service is not busy enough to benefit from a CP program	Type of community served	< 0.001†
Willingness to volunteer for additional education to become a CP	Age	0.03‡
Willingness to volunteer to attend additional CP education	Years of EMS experience at current level of EMS certification/licensure	0.01‡

*CP*, community paramedic; *EMS*, emergency medical services.

* Mann-Whitney *U* test.

† Kruskal-Wallis one-way analysis of variance.

‡ Pearson product-moment coefficient of correlation.

**Table 5:** Logistic regression model results for willingness to perform CP duties.

Parameter	OR	p-value	95% CI
Gender	4.651	0.03	1.186, 18.236
Race	0.191	0.02	0.049, 0.744
Perceived CP hours on duty	0.198	<0.001	0.087, 0.449
Constant	6.124	<0.001	

*OR*, odds ratio; *CI*, confidence interval; *CP*, community paramedic.
