# Supplementary material for: Emergency Medical Services Professionals’ Attitudes About Community Paramedic Programs
Source: West J Emerg Med. 2017 May 1;18(4):630–9. doi: 10.5811/westjem.2017.3.32591 (PMC5468069; doi:10.5811/westjem.2017.3.32591)
Supplement: Supplementary file 1 [file wjem-18-630-s001.docx]

Survey #001

**Section 1**

Please complete all demographic and EMS professional information.

| 1. |  | I am:  male  female | |  |
| --- | --- | --- | --- | --- |
|  |  |  | |  |
| 2. |  | Age: | |  |
|  |  |  | |  |
| 3. |  | I am: | |  |
|  |  | African American/Black | |  |
|  |  | Asian | |  |
|  |  | Hispanic | |  |
|  |  | American Indian or Alaska Native | |  |
|  |  | White | |  |
|  |  | Multi-cultural | |  |
|  |  | Other | |  |
|  |  |  | |  |
| 4. |  | Education completed: | |  |
|  |  | High school or GED | |  |
|  |  | Some college | |  |
|  |  | Associate Degree | |  |
|  |  | Bachelor’s Degree | |  |
|  |  | Master’s Degree | |  |
|  |  | Doctoral Degree | |  |
|  |  |  | |  |
|  |  |  | |  |
| 5. |  | Total years of EMS experience (including **all** EMS levels) | |  |
| 6. |  | Current Level of EMS certification/licensure | |  |
|  |  | Emergency Medical Responder/First Responder | |  |
|  |  |  | Emergency Medical Technician/EMT-B | |
|  |  |  | Advanced EMT/EMT-Intermediate | |
|  |  |  | Paramedic/EMT-P | |
|  |  |  | |  |
| 7. |  | Years of EMS experience at current level of EMS certification/licensure | |  |
|  |  |  | |  |
| 8. |  | How many runs do you currently average in a typical shift? | |  |
| 9. |  | Which of the following choices **best** describes your typical shift? (check the **one** most accurate answer) | |  |
|  |  | Days (8-12 hour shifts) | |  |
|  |  | Afternoons/ Evenings (8-12 hour shifts) | |  |
|  |  | Nights (8-12 hour shifts) | |  |
|  |  | 24 hours | |  |
|  |  | More than 24 hours  Other | |  |

| 10. |  | With what type of EMS service are you associated? |
| --- | --- | --- |
|  |  | (check only the **primary** service if there is more than one) |
|  |  | Fire Department |
|  |  | Hospital-based |
|  |  | Private Service |
|  |  | Public Utility/nonprofit |
|  |  | Third Service/government/county |
|  |  | Other |

| 11. |  | How would you describe the community in which **most** of your EMS work time is spent? |
| --- | --- | --- |
|  |  | Rural (<2,500 people) |
|  |  | Small Town (2,500-74,999) |
|  |  | Large Town (75,000-149,000) |
|  |  | Mid-sized City (150,00-500,000) |
|  |  | Suburb of mid-sized city |
|  |  | Large City (500,000 or more) |
|  |  | Suburb/fringe of large city |

12. Does your service currently utilize a Community Paramedic model to provide patient care?

Yes

No

13. What is your current rank at your EMS service? (Check the **one** most accurate answer that reflects a majority of your current time on duty)

Field provider of patient care

Supervisor/management

Other

14. What are the current number of hours per shift/day worked in which you think you could commit to a Community Paramedic program?

0 hours

1 hour

2 hours

3 hours

4 hours

More than 4 hours

15. Which of the following types of patient education programs would be most beneficial for a Community Paramedic program to develop and implement? (Indicate all that apply)

Congestive Heart Failure

Diabetes

Hypertension (high blood pressure)

Cardiac Rehab (post myocardial infarction/heart attack)

Fall Prevention

Other ________________

16. In your opinion, what is the percentage (%) of the patients you currently encounter in the field that would benefit from a Community Paramedic program?

     %

**Section 2**

Each question in this section refers to ***your*** views on a Community Paramedic program.

1. I currently have a good understanding of a Community Paramedic program.

| **Strongly**  **Disagree** | -3 | -2 | -1 | 0 | +1 | +2 | +3 | **Strongly**  **Agree** |
| --- | --- | --- | --- | --- | --- | --- | --- | --- |

2. I would volunteer to attend additional education to become a Community Paramedic.

| **Strongly**  **Disagree** | -3 | -2 | -1 | 0 | +1 | +2 | +3 | **Strongly**  **Agree** |
| --- | --- | --- | --- | --- | --- | --- | --- | --- |

3. A Community Paramedic program will help those in most need (i.e. the very young, the very old, and the disabled).

| **Strongly**  **Disagree** | -3 | -2 | -1 | 0 | +1 | +2 | +3 | **Strongly**  **Agree** |
| --- | --- | --- | --- | --- | --- | --- | --- | --- |

4. A Community Paramedic program should be a significant responsibility for EMS in my community.

| **Strongly**  **Disagree** | -3 | -2 | -1 | 0 | +1 | +2 | +3 | **Strongly**  **Agree** |
| --- | --- | --- | --- | --- | --- | --- | --- | --- |

5. I would perform the duties of a Community Paramedic with as much or more enthusiasm as I currently have for traditional, prehospital patient care.

| **Strongly**  **Disagree** | -3 | -2 | -1 | 0 | +1 | +2 | +3 | **Strongly**  **Agree** |
| --- | --- | --- | --- | --- | --- | --- | --- | --- |

6. My coworkers would be in favor of performing Community Paramedic duties.

| **Strongly**  **Disagree** | -3 | -2 | -1 | 0 | +1 | +2 | +3 | **Strongly**  **Agree** |
| --- | --- | --- | --- | --- | --- | --- | --- | --- |

7. The community I serve would be in favor of our service performing Community Paramedic duties.

| **Strongly**  **Disagree** | -3 | -2 | -1 | 0 | +1 | +2 | +3 | **Strongly**  **Agree** |
| --- | --- | --- | --- | --- | --- | --- | --- | --- |

8. The leaders in my EMS service, in general, would support our organization’s involvement in a Community Paramedic program.

| **Strongly**  **Disagree** | -3 | -2 | -1 | 0 | +1 | +2 | +3 | **Strongly**  **Agree** |
| --- | --- | --- | --- | --- | --- | --- | --- | --- |

9. I became an EMS professional in order to save lives during emergencies - not to participate in a Community Paramedic program.

| **Strongly**  **Disagree** | -3 | -2 | -1 | 0 | +1 | +2 | +3 | **Strongly**  **Agree** |
| --- | --- | --- | --- | --- | --- | --- | --- | --- |

10. My EMS service is not busy enough to benefit from a Community Paramedic program.

| **Strongly**  **Disagree** | -3 | -2 | -1 | 0 | +1 | +2 | +3 | **Strongly**  **Agree** |
| --- | --- | --- | --- | --- | --- | --- | --- | --- |

11. My EMS service is too understaffed to develop a Community Paramedic program.

| **Strongly**  **Disagree** | -3 | -2 | -1 | 0 | +1 | +2 | +3 | **Strongly**  **Agree** |
| --- | --- | --- | --- | --- | --- | --- | --- | --- |

12. Performing Community Paramedic duties would take up valuable down-time that I depend upon (i.e. for rest and other personal activities) while on duty.

| **Strongly**  **Disagree** | -3 | -2 | -1 | 0 | +1 | +2 | +3 | **Strongly**  **Agree** |
| --- | --- | --- | --- | --- | --- | --- | --- | --- |

13. I work hours that would not be compatible with Community Paramedic duties for many people. (i.e., most people would not be interested in a 3:00 a.m. visit from a Community Paramedic in their home.)

| **Strongly**  **Disagree** | -3 | -2 | -1 | 0 | +1 | +2 | +3 | **Strongly**  **Agree** |
| --- | --- | --- | --- | --- | --- | --- | --- | --- |

14. My EMS service would be willing to develop a specific position or positions dedicated to performing Community Paramedic duties.

| **Strongly**  **Disagree** | -3 | -2 | -1 | 0 | +1 | +2 | +3 | **Strongly**  **Agree** |
| --- | --- | --- | --- | --- | --- | --- | --- | --- |

Thank you for your participation!
